# Editorial: Electrospinning of Bioinspired Materials and Structures for Bioengineering and Advanced Biomedical Applications

**DOI:** 10.3389/fbioe.2021.739613

**Published:** 2021-09-06

**Authors:** Luca Cristofolini, Maria Letizia Focarete, Seeram Ramakrishna, Alberto Sensini, Andrea Zucchelli

**Affiliations:** ^1^ Department of Industrial Engineering, Alma Mater Studiorum-Università, Bologna, Italy; ^2^ Health Sciences and Technologies – Interdepartmental Center for Industrial Research (CIRI-HST), Alma Mater Studiorum-Università, Bologna, Italy; ^3^ Department of Chemistry “G. Ciamician”, Alma Mater Studiorum-Università, Bologna, Italy; ^4^ Department of Mechanical Engineering, Center for Nanofibers and Nanotechnology, National University of Singapore, Singapore, Singapore; ^5^ Applications in Mechanical Engineering and Materials Technology – Interdepartmental Center for Industrial Research (CIRI-MAM), Alma Mater Studiorum-Università, Bologna, Italy

**Keywords:** electrospinning, bioinspired structures, biomaterials, bioengineering, advanced biomedical applications

The Research Topic “*Electrospinning of Bioinspired Materials and Structures for Bioengineering and Advanced Biomedical Applications*” includes submissions that relate to the “Biomaterials” and “Bionics and Biomimetics” sections of Frontiers in Bioengineering and Biotechnology. The collection aims to provide an overview of how electrospinning, inspired by nature, can reproduce the hierarchical structure and biomechanical properties of biological tissues, ranging from the nanoscale to the macroscale. The development of such innovative nanofibrous structures requires the improvement of both functionalization and biofabrication strategies, to enhance the scaffold bioactivity and to drive cells in the regeneration of the extracellular matrix (ECM) of the target tissues of interest. Recent technological advances have given rise to the availability of intelligent and smart biomaterials for the regeneration of innovative procedures for manufacturing nanometric structures, and methods for assembling multiscale hierarchical structures. Furthermore, imaging has improved considerably in the last few years, allowing multimodal imaging with nanometric resolution.

The study by Daraeinejad and Shabani targeted the regeneration of electroactive tissues. They investigated the most suitable solvent system for electrospun conductive polyaniline (PANI). To enhance the spinnability of PANI, they electrospun the polymer in a blend with poly (ether sulfone) (PES), using HFIP as a solvent. These nanofibers have been shown to enhance fibroblast proliferation and elongation after 7 days of culture.

Delaine-smith et al. investigated how the orientation of gelatin-coated poly (caprolactone) nanofibers (from random to aligned) can influence mature murine osteoblasts or human osteogenic mesenchymal progenitors in the production of mineralized collagenous matrix and its mechanical properties. Murine osteoblasts deposited more calcium-containing matrix on aligned scaffolds, while human osteogenic mesenchymal progenitors exhibited more collagen and calcium deposition on randomly orientated ones. These results suggested that a suitable scaffold for mesenchymal stem cell osteogenesis, to enhance differentiation and control the organization of the ECM, needs to combine random and oriented nanofibers.

The functionalization of poly (3-hydroxybutyrate-co-4-hydroxybutyrate) [P (3HB-co-4HB)] copolymer nanofibrous scaffolds for cardiac tissue engineering was explored by Vigneswari et al. The P (3HB-co-4HB) copolymer was electrospun by obtaining random nanofibrous mats functionalized with RGD peptides. The scaffolds promoted the attachment and proliferation of H9c2 cells.

Giuntoli et al. aimed to improve the tissue integration of meshes for abdominal hernia surgery. They explored an electrospun nanofibrous coating to speed up cell attachment and tissue formation. A commercial PP hernia mesh was coated with a nanofibrous membrane from a polycaprolactone and gelatin blend. Human fibroblasts adhered to the electrospun substrate without necrosis induction, suggesting its suitability for abdominal wall hernia repair.

Hiraki et al. optimized electrospun nanofibers loaded with superparamagnetic iron oxide nanoparticles (SPIONs), to magnetically promote the orientation of mouse tenocytes and human epithelial cells. SPION density and magnetic field, guided and tuned fiber alignment. The functionalization of microfiber segments with cell adhesive peptides induced a uniaxial alignment of tendon fibroblasts. The authors also demonstrated that these fibrillar hydrogel composites were able to drive multicellular migration from human epithelial cell spheroids, promoting the separation of single cells.

Sensini et al. aimed to mimic the mechanical behavior of natural tendon fascicles at physiological strain levels. They produced prosthetic fascicle-inspired electrospun nylon 6,6 bundles with different collector peripheral speeds, reaching different orientations of nanofibers (from randomly to mostly axially aligned arranged). For each sample category, the transition (end of toe region) and the inflection (switch from strain-stiffening to strain-softening) points of the stress-strain curves were calculated. This study showed that these points can shift with nanofiber arrangement and compare their values with those of tendons.

A review on these topics is included (Sensini et al.), covering the state of the art of electrospinning strategies adopted to mimic the tendon/ligament-to-bone insertions (enthesis) and the myotendinous junction. The most relevant papers in the field were described following the increasing hierarchical complexity of scaffolds. The biofabrication strategies adopted, the *in vitro* and *in vivo* outcomes, the ECM production, and the biomechanical performances were described, highlighting the strengths and open questions of interfacial tissue engineering.

This collection of articles demonstrates how electrospinning is able, by tailoring the organization of its nanofibers and their functionalization, to reproduce the structure and mechanics of the ECM of human tissues at different levels of hierarchical complexity, offering biomimetic environments in which the tissue of interest is proliferated and regenerated. However, the analysis undertaken by the papers in this Research Topic does also reveal unsolved challenges, such as the possibility to effectively ensure the multi-tissue regeneration of electrospun scaffolds by guiding stem cell fate and successfully reproduce a faithful multiscale hierarchical structure of the target tissue of interest.

